# Rice straw biochar as a novel niche for improved alterations to the cecal microbial community in rats

**DOI:** 10.1038/s41598-018-34838-1

**Published:** 2018-11-06

**Authors:** Jie Han, Jun Meng, Shuya Chen, Chuang Li, Shuo Wang

**Affiliations:** 1 Key Laboratory of Zoonosis of Liaoning Province, College of Animal Science and Veterinary Medicine, Shenyang Agricultural University, Dongling Road 120, Shenyang, Liaoning Province 110866 P.R. China; 2 Liaoning Biochar Engineering & Technology Research Center, Shenyang Agricultural University, Dongling Road 120, Shenyang, Liaoning Province 110866 P.R. China; 3 Testing and Analysis Center, Shenyang Agricultural University, Dongling Road 120, Shenyang, Liaoning Province 110866 P.R. China

## Abstract

Biochar as additive has been shown positive effect in animal production, which may be linked to the role of gastrointestinal microbial modulation. This study aimed to assess the effects of biochar on the gut microbial communities in terms of their structure and diversity. Illumina high-throughput technology was utilized to evaluate the cecal microbial community in Wistar rats received oral rice straw biochar (RSB) at 1120 mg/kg of body weight for 5 weeks. RSB improved the gut mucosal structure and epithelial integrity. More importantly, principal coordinate analysis of UniFrac distances based on a 97% operational taxonomic unit composition and abundance indicated that the bacterial community was ameliorated after RSB addition (*P* < 0.05). *Firmicutes* and *Bacteroidetes* were found to be the prevalent phyla accounting for approximately 90% of the sequences and their ratio of relative abundance was increased by RSB addition (*P* < 0.05). Improved bacterial proportion of unclassified *Lachnospiraceae* (*P* < 0.001), *Oscillibacter* (*P* = 0.02), and *Clostridium IV* (*P* = 0.02) and *XIVa* (*P* = 0.02) as well as decreased abundances of *Prevotella* (*P* < 0.001) and *Bacteroides* (*P* = 0.03) were also detected at genus level following RSB treatment. These results revealed that RSB altered and improved the cecal microbial community, which may contribute to the affected growth and gut status in rats.

## Introduction

The animal gut is the natural habitat for a large and dynamic bacterial community. The relevance and effects of the endogenous microflora on host physiology and pathology have been well documented^1^ from nutritional status to behavior and stress response, that ultimately affect health status^2,3^. Diet is generally accepted to be a major influence on the gut microbial community. Alterations in the structure and diversity of the gut microbial community can occur with long-term consumption of a habitual diet^4^ as well as short-term consumption of certain diets^5^; such alterations to the gut microbial profile may be temporal^5^ or irreversible^6^.

Carbonaceous adsorbents are a large group of natural substances generally prepared from plant residues and characterized primarily by adsorption property based on their inhomogeneous microporous structure that contribute to increased oxidation resistance and provide applicable habitats for microorganisms^7^. In several management regimes, carbonaceous adsorbents are recommended as an additive^8^, and it has achieved a great of positive effects in livestock production including improvement of growth performance^9^, modulation of the intestinal morphology^10^, microflora counts^11,12^, and reduction of gut noxious gas emission^9^, and the ability to enhance immune response^11^.

Biochar is a novel type of carbonaceous adsorbent not only in terms of the prepared materials from agricultural wastes such as crop straw, husks, shell, and others, but also its preparation by thermal degradation of organic matters in environments where oxygen is absent or limited conditions. This production engineering make biochar has larger surface area and macropores due to the loss of volatiles during pyrolysis to provide more spacious sites for microorganisms to multiply^13^. In China, biochar may act as a novel niche for animal additive in respect to unlimited sources of raw materials, competitive prices, friendly environmentally production process, and our report has suggested the abilities of rice straw biochar (RSB) to enhance nutrient digestibility, lead to better growth performance and improve immune responses in piglets^14^. The aforementioned affected issues may be linked to the regulation of gastrointestinal microorganism under biochar application. However, little information is understood about the effects of biochar on gastrointestinal microbial community although the relative effect in soil^15^ and composting^16^ has been well documented.

In the present study, the growth performance, intestinal epithelial integrity, and structure and diversity of bacterial communities of cecum in rat were examined. We focused on determining the impact of RSB on the gastrointestinal microbial community by illumina high-throughput technology to preliminarily understand the reason that affected growth and intestinal status by biochar.

## Results

### The effects of RSB on BW and intestinal morphology

**Figure 1:**
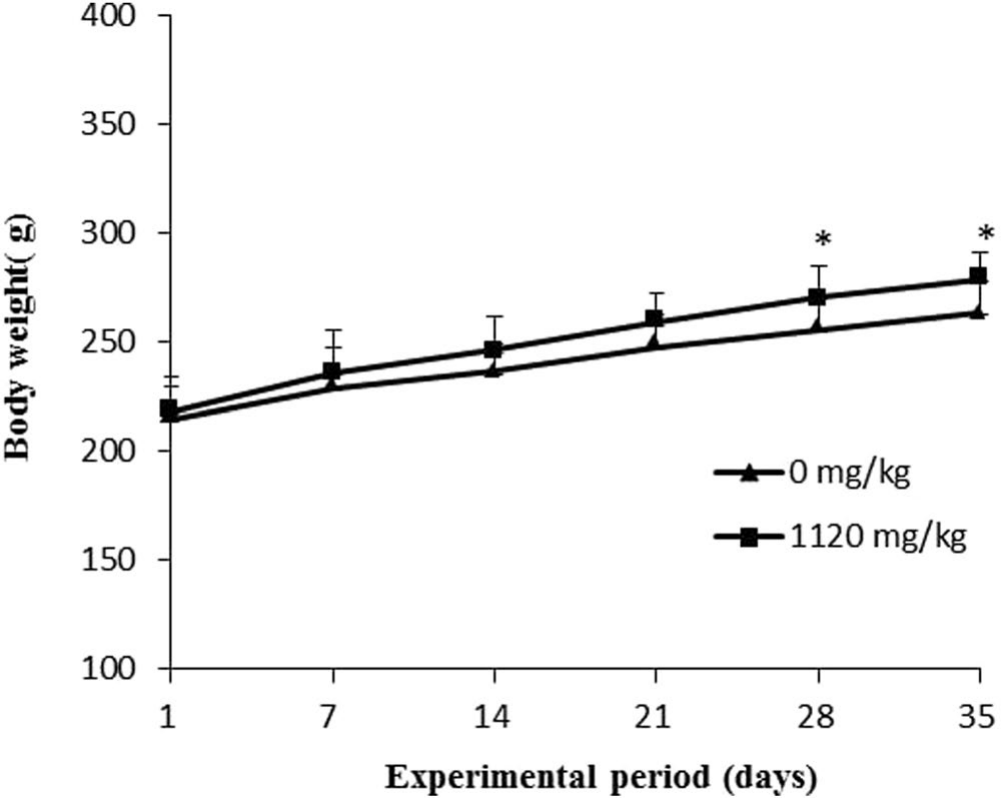
Body weight in rats treated with RSB for 5 weeks. Values are presented as the mean ± SD (n = 10). *Indicates a significant difference from the control group (*P* < 0.05).

**Figure 2:**
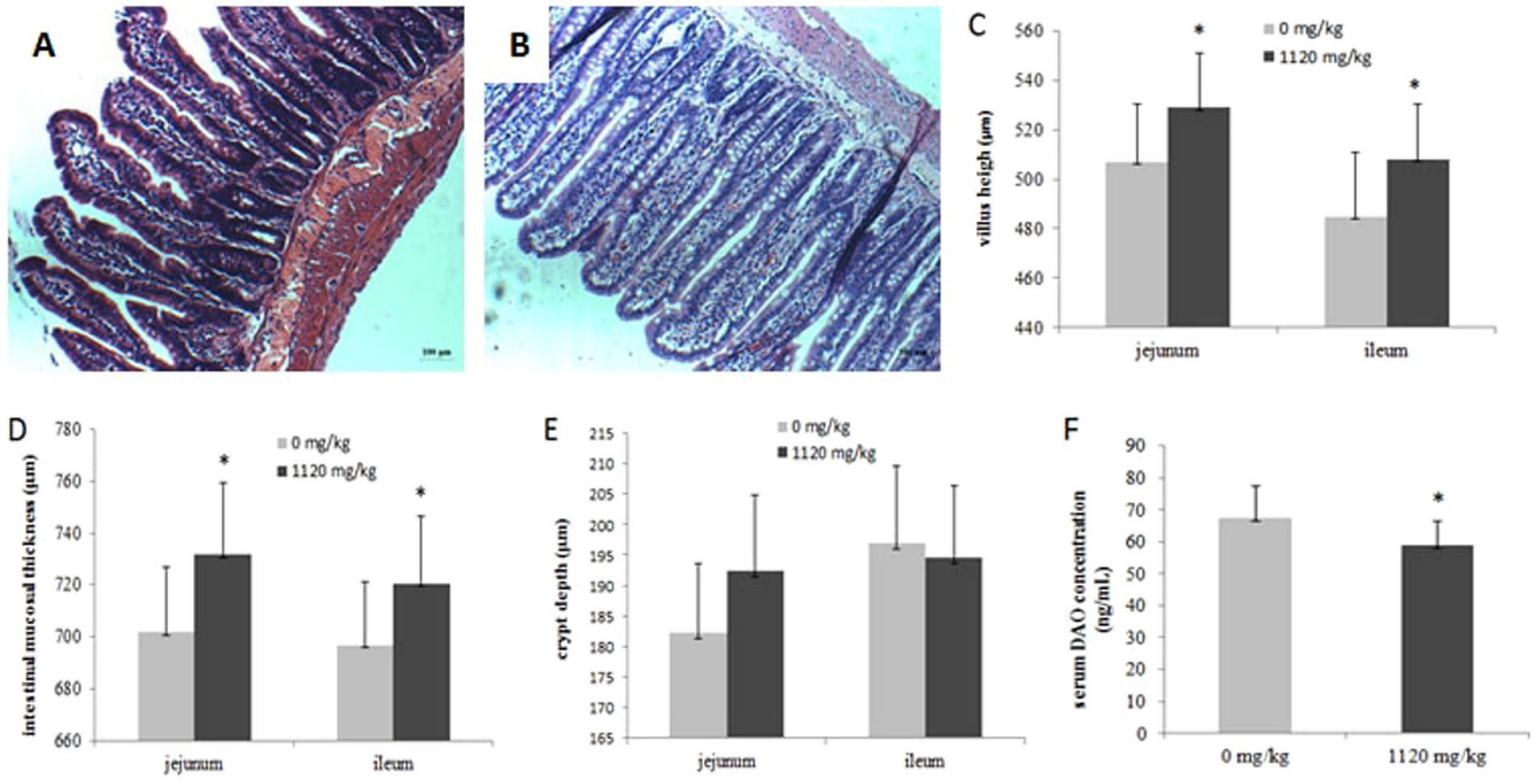
Effects of RSB administration on gut villus morphology. Values are presented as the mean ± SD. (**A,B**) Representative images of H&E staining for gut villi in the ileal tissue (n = 5); (**C**) intestinal mucosal villus height in the jejunum and ileum (n = 5); (**D**) intestinal mucosal thickness in the jejunum and ileum (n = 5); (**E**) intestinal crypt depth in the jejunum and ileum (n = 5); (**F**) serum DAO concentration in rats (n = 10). *Indicates a significant difference from the control group (*P* < 0.05).

RSB treatment resulted in a significant increase in rat BW at the end of weeks 4 and 5 (*P* < 0.05) (Fig. 1). Representative images of H&E staining for ileal villus morphology and intestinal villus parameters are presented in Fig. 2. Ileal mucosal histological examination in the RSB group demonstrated a clear intestinal wall and intact intestinal epithelium structure without hyperemia and inflammatory infiltration (Fig. 2A,B). As expected, compared with the control group, the oral RSB treatment group showed significantly increased villus height in the jejunum and ileum by 4.30% and 4.81% (*P* < 0.05) (Fig. 2C), respectively, as well as increased mucosal thickness by 4.26% and 3.40% (*P* < 0.05) (Fig. 2D), respectively; no effect on crypt depth was observed following RSB treatment (*P* > 0.05) (Fig. 2E). Determination of serum DAO concentrations by ELISA revealed decreased serum DAO levels in the RSB group (*P* < 0.05) (Fig. 2F).

### Intestinal epithelium ultra-structure profile in response to oral RSB

The ultra-structure of the intestinal epithelium was evaluated by TEM to determine the influence of RSB on tight junction (TJ) structures and epithelial internal organelles, as shown in Fig. S1. Compared with the rats in the control group, those in the RSB group exhibited a more orderly arrangement and longer microvilli in the ileum (Fig. S1A,B). Similarly, the application of RSB led to more rounded nuclei in the intestinal epithelium, denser chromatin, and more mitochondria with larger volumes (Fig. S1C,D). TJs in the ileum in the RSB group exhibited more intact structures and electron-dense material between adjoining cells (Fig. S1E,F), and the TJ width was decreased by 11.49% in the RSB group compared with that in the control group (*P* < 0.05) (Fig. S1G).

### Pyrosequencing overview and community β-diversity

High-throughput sequencing was used to determine the effect of RSB on gut microbial communities. More than 800,000 valid reads produced from 13 cecal content samples with 67,640 sequences per sample were shown in Table S1 (anomalous sample C1 in control group has been identified and removed during library preparation), and these high-quality sequences were then clustered into OTUs according to a cut-off of 97% sequence similarity. The OTUs ranged from 849 to 1937 per sample, with an average of 1337 OTUs per sample and a total of 17382 OTUs in all samples (Table S1).

**Figure 3:**
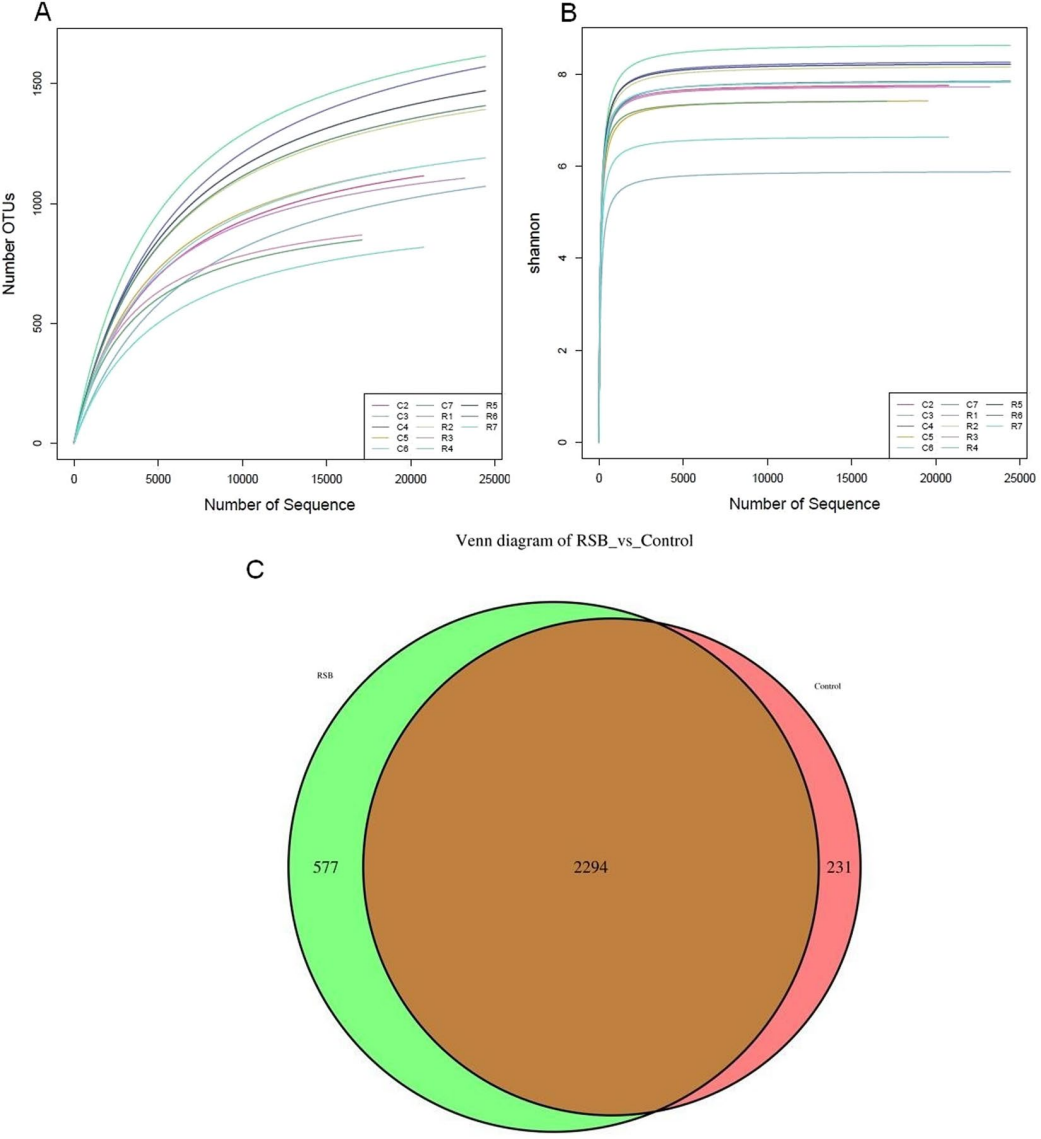
Diversity and richness of the gut microbiota in rats. (**A**) Number of OTUs; (**B**) Number of Sequences; (**C**) OTUs Venn diagram.

Rarefaction curve, Shannon index, Simpson index, and Chao1 index analyses were performed to assess the diversity of microbial species per sample (Table 1, Fig. 3). Rarefaction curve analysis demonstrated that the curve tended to gradually smooth, exceeding a sequencing quantity of 15,000, indicating that the sequencing depth basically fulfilled the needs of the experimental analysis and that most of the cecal microbes in each sample were captured under this condition (Fig. 3A). Chao1 estimations ranged from 1026.69 to 1882.77, reflecting the ecological species richness of the bacterial community and primarily highlighting species amounts. The value of the Shannon index ranged from 5.87 to 8.72, representing and correlating positively with the distribution evenness of different species. Simpson values indicate the probability of individuals belonging to different species in a community and correlate negatively with species richness. The average values of the Chao1 and the Shannon indices in the RSB group were greater than those in the control group (Fig. 3B), while the reverse was true for the Simpson parameter (Table 1). Meanwhile, Venn diagrams indicated that there were 2294 common OTUs between the two groups, and the counts of specific OTUs increased from 231 in the control group to 577 in the RSB group (Fig. 3C).

**Table 1:** Diversity estimation of the 16S rRNA gene libraries of the cecal content of rats following RSB treatment. C2~C7 indicate rats from control group; R1~R7 indicate rats from RSB group.

Sample name	OTU number	Chao1	Shannon	Simpson
C2	1161	1338.91	7.76	0.99
C3	1101	1352.01	5.87	0.88
C4	1937	1882.77	8.72	0.99
C5	1178	1422.27	7.44	0.97
C6	849	1026.69	6.63	0.97
C7	877	1162.23	7.42	0.98
R1	1140	1308.49	7.71	0.99
R2	1780	1713.22	8.13	0.99
R3	912	1170.68	7.74	0.99
R4	1756	1865.29	8.62	0.90
R5	1829	1754.30	8.20	0.99
R6	1568	1664.62	7.85	0.98
R7	1294	1451.15	7.84	0.99
Total	17382			
Average	1337.08

### The unweighted pair group method with the arithmetic mean (UPGMA) and PCoA of the cecal microbial structure

**Figure 4:**
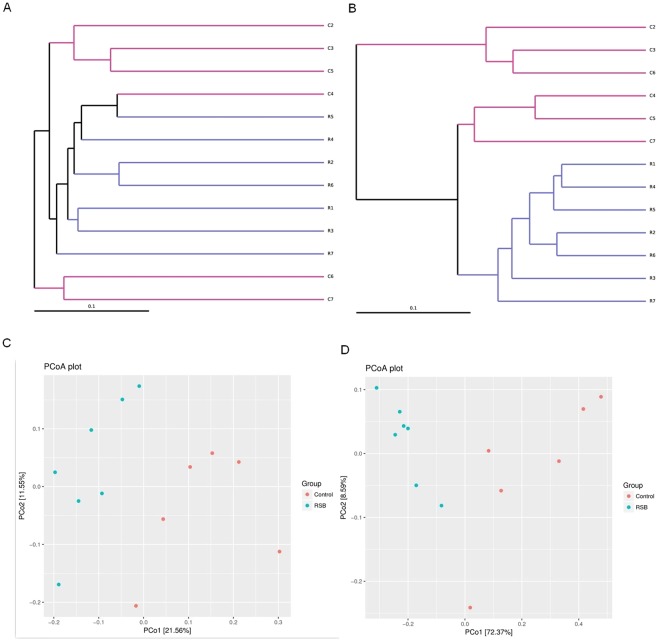
Responses of the gut microbiota structure to RSB treatment. (**A,B**) The UPGMA of the cecal microflora in rats, (**A**) unweighted UniFrac; (**B**) weighted UniFrac; (**C,D**) PCoA score plot based on the (**C**) unweighted and (**D**) weighted UniFrac metrics.

The UPGMA based on the weighted UniFrac and unweighted UniFrac analysis is presented in Fig. 4. The means of the unweighted UniFrac considering only the detected species diversity showed that the samples clustered well within the two groups in addition to samples 6 and 7 in the control group (Fig. 4A). Meanwhile, the weighted UniFrac analysis showed that RSB exhibited a distinctive effect on the cluster when integrating both the diversity and abundance of the microbiota (Fig. 4B). UniFrac distance-based PCoA was employed to assess the structural changes in the gut microbiota induced by oral RSB. Both unweighted and weighted UniFrac analyses showed overt changes in the overall gut microbial community in response to RSB administration, with a greater distinction obtained by unweighted UniFrac analysis compared with that by the weighted assessment (Fig. 4C,D).

### Microbial community composition analysis

**Figure 5:**
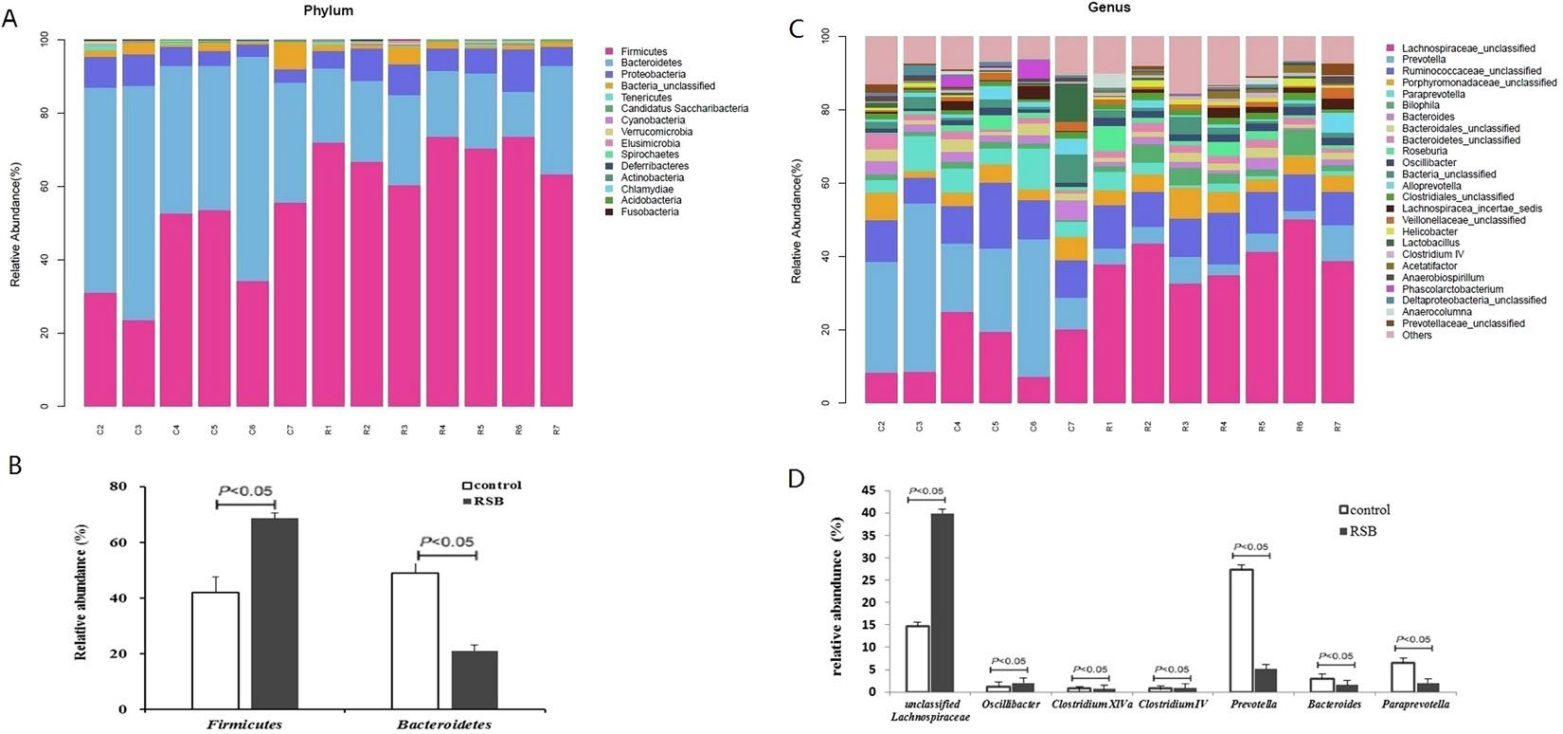
Cecal bacterial community composition at different taxonomic levels between control and RSB groups. (**A**) Relative abundance of gut microbiota detected at phylum level; (**B**) Relative abundance of gut microbiota detected at genus level; (**C**) Relative abundance of affected significantly bacterial taxa by RSB treatment at genus level; (**D**) Relative abundance of affected significantly bacterial genus by RSB treatment.

The taxa summary revealed distinctive changes in the gut microbial composition in response to RSB administration. Our results at the phylum level (Fig. 5A) indicated that 15 phyla were detected in all samples, and *Firmicutes*, *Bacteroidetes*, *Proteobacteria*, and *Bacteria unclassified* were the dominant taxa with relative abundances of 41.79% vs 68.61%, 48.89% vs 21.01%, 5.63% vs 7.46%, and 2.71% vs 1.79% in the control and RSB groups, respectively. More importantly, *Firmicutes* in the RSB groups was markedly increased by 64.18% (*P* < 0.05), whereas *Bacteroidetes* (21.01%) was decreased by 57.03% (*P* < 0.05) compared with those in the control group (Fig. 5B).

At the genus level, we also detected striking changes in the gut microbial composition in the RSB group (Fig. 5C). Our results indicated that 122 genera were detected in all samples, and unclassified *Lachnospiraceae*, *Prevotella*, *Ruminococcaceae*, and unclassified *Porphyromonadaceae* were the dominant taxa with proportions of 14.64% vs 39.92%, 27.38% vs 5.14%, 11.30% vs 10.90%, and 4.5% vs 5.11% in the control and RSB groups, respectively. Above all, unclassified *Lachnospiraceae*, *Oscillibacter*, *Clostridium XlVa*, and *Clostridium IV* in the RSB group were markedly increased by 172.68% (*P* < 0.05), 67.48% (*P* < 0.05), 436.36% (*P* < 0.05), and 117.5% (*P* < 0.05), respectively, whereas *Prevotella*, *Bacteroides*, and *Paraprevotella* were all decreased by 81.23% (*P* < 0.05), 46.60% (*P* < 0.05), and 69.54% (*P* < 0.05), respectively, compared to the control group (Fig. 5D).

## Discussion

The present study demonstrated that oral RSB positively improved the morphological appearance of gut mucosa in rats using H&E staining. Additionally, RSB elevated the mucosal integrity, as supported by the amelioration of electron-dense materials and TJ width between adjoining cells determined using TEM and a decline in the serum DAO concentration. DAO is an intracellular enzyme with high activity that exists in intestinal villous cells and declines when IEC is injured, reflecting changes in intestinal mucosal permeability^17^. Collectively, the aforementioned results suggested that RSB administration enhanced intestinal integrity in rats.

Sequences were assigned to OTUs based on the similarity at a threshold of 97% to analyze the bacterial community structure as well as subsequent abundance and diversity^18^. After removing the noise and low-quality sequences, we determined that there were 1183 to 1468 OTUs per sample and 231 to 577 OTUs specific to the RSB and control groups, indicating that sustained RSB induced more specific flora OTUs. Proper biodiversity estimation is crucial for understanding the structure, function, and evolution of microbial communities^18^. The Shannon (mean value of 8.01 vs 7.31) and Chao1 indices (mean value of 1561.01 vs 1364.15) in the RSB group were much higher than those in the control group, indicating that RSB treatment increased the diversity of the gut bacterial community in rats, which was further confirmed by similar Simpson index results, although the values were slightly different (mean value of 0.989 vs 0.963).

Past studies have revealed that gut microbiota in mammalian^19^, cattle^20^ and swine^21^, are dominated by two bacterial phyla, *Bacteroidetes* and *Firmicutes*, with other phyla including *Actinobacteria, Proteobacteria, Fusobacteria* and *Verrucomicrobia*^19,22^ at subdominant levels, which are in line with our study demonstrating that there were 13 bacterial phyla following RSB treatment with the most abundant phyla present of *Firmicutes* and *Bacteroidetes* constituting approximately 90% of the total cecal bacteria detected. *Firmicutes* as the largest portion of gut microbiome in our research, the metabolic end-products of some species in this phylum have been shown to be involved in energy metabolism and the development of the intestinal epithelium^20,22^, and the members of the diverse bacterial phylum *Bacteroidetes* play a beneficial role in the degradation of organic matter^23^, especially in the gastrointestinal tract, which may be responsible for the aforementioned improvement of intestinal integrity. More importantly, the application of RSB in our study significantly increased the relative abundance of *Firmicutes* by 68.61% and decreased *Bacteroidetes* by 57.03%, consequently increasing the ratio of *Firmicutes* to *Bacteroidetes*. The change in the ratio has been considered to contribute to the health benefits in many researches^24,25^ and hence partially suggested that RSB exerted its beneficial effects on growth and intestinal health within the gut microbial environment.

The diversity of gut microbiome at genus level exhibited notable differences with RSB addition. The elevated proportions of major bacteria genera of Unclassified *Lachnospiraceae*, *Clostridium IV* and *XIVa*, and *Oscillibacter* belonging to the gram-positive phyla of firmicutes accounted for approximately 40% in cecal microbiota after RSB addition. *Oscillibacter* was significantly found in more samples from healthy control test subjects than from patients diagnosed with Crohn’s disease or hypertension^26,27^, which may be a symbol of healthy status of rats after receiving RSB. *Lachnospiraceae* and *Clostridium IV* and *XIVa* as the dominant genera of cecal microbiota in RSB group can degrade complex polysaccharides to short-chain fatty acids including acetate, butyrate, and propionate, which can be used for energy by the host^28,29^, and forms a barrier against the invasion of pathogenic bacteria due to pH reduction. Interestingly, *Clostridium XIVa* also exhibits mucin adhesion activity and thus reduces mucin utilization by intestinal pathogens^30^. The promoted aforementioned species by RSB inclusion sustained intestinal environment improvement and energy metabolism modulation and resulted in weight gain. Further metabolite detection in the cecum is needed to determine if the alterations in short-chain fatty acids benefited from RSB treatment.

Meanwhile, the decrease in the proportion of the major species included *Prevotella* and *Bacteroides* were also detected after RSB addition. *Prevotella* strains are often linked to chronic inflammatory conditions^31^ and *Bacteroides* are opportunistic human pathogens, causing infections of the peritoneal cavity and requiring gastrointestinal surgery due to abscess formation^32^ although these species sometimes can benefit their host by excluding potential pathogens from colonizing the gut^32^. The changed proportion of *Prevotella* and *Bacteroides* suggested that RSB inclusion may reduce certain opportunistic pathogens and improve intestinal inflammatory conditions.

**Figure 6:**
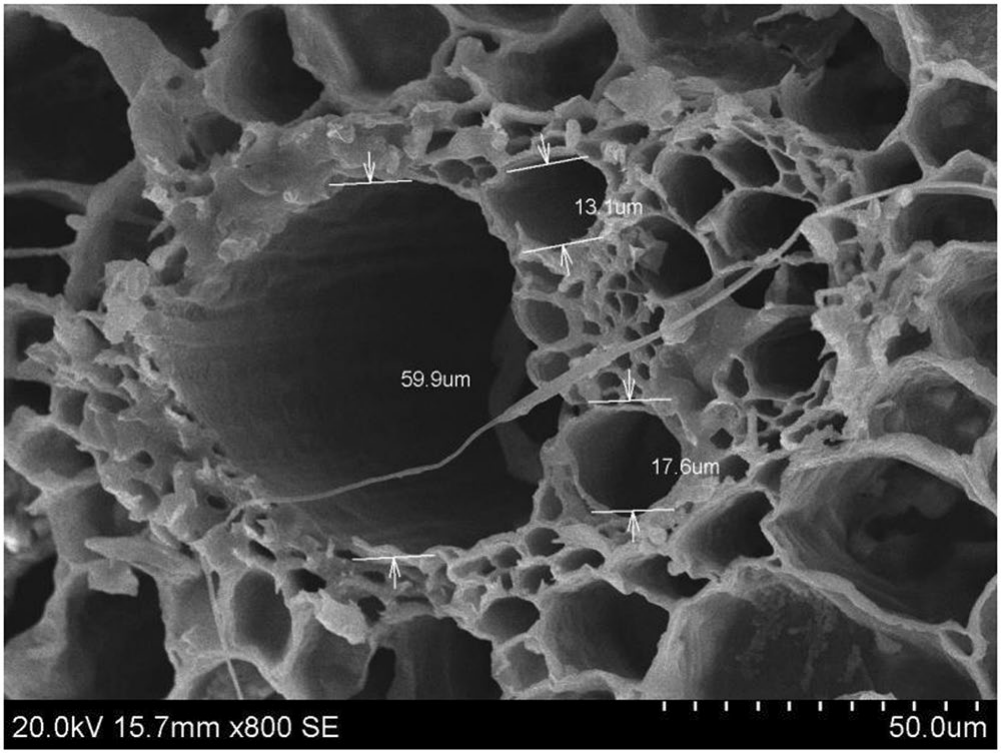
TEM image of RSB with arrows indicating the diameter of micropores.

The ability of biochar to modulate microorganisms in terms of microbial activity, abundance and community have been approved in the many applications in soil^15,33,34^ and composting^16,35^, depending on the intrinsic properties such as larger surface area, macropores, physico-chemical parameters, and available nutrients. The possible mechanism of the improvement in gut community under biochar application in our study may be elaborated as followed: RSB used here as a slow-pyrolysis product with many macropores (>50 nm) is optimal carrier for bacterial inoculum^36^ (Fig. 6) because the optimal pore size of biochar should be 2–5 times larger than cell size^37^. It can provide more spacious sites and nutrition for some of microorganisms to multiply or act as a “refuge” to prevent hurt from intestinal hazard factors. Another possibility may be the changes in physico-chemical properties of gut content directly or indirectly in terms of water content, pH and nutrition induced by biochar and intestinal microorganisms are strongly sensitive to the environmental variables^38^. Our findings supported aforementioned inference to a certain extent that the increased proportion of some species such as *Lachnospiraceae* and *Clostridium IV* and *XIVa* could metabolize to produce short chain fatty acids after RSB addition, which affected conversely intestinal bacteria through pH alteration. In addition, we surmised that RSB may benefit the growth of some microbes that can use the inherent carbon sources of RSB and result in selective pressure on the structure of intestinal bacterial community, which was supported by the notable change in abundance of *Firmicutes* in our study due to its genetic capacity devoting to the utilization of a variety of carbon sources including many plant-derived molecules^39^. Further study should be carried out to detect aforementioned hypothesis by checking the proliferation and the carbon utilization capacity of the affected microbial genera by RSB *in vitro*.

In conclusion, our findings firstly revealed that RSB addition profoundly altered and improved the cecal microbial structure and diversity in rats by 16S rRNA gene high-throughput sequencing, which may contribute to subsequent improvement of gut integrity and growth. Further work involving the proliferation of affected microbe *in vitro* should be conducted to discuss profoundly the mechanism of gastrointestinal microbial modulation by RSB.

## Materials and Methods

### RSB preparation and characterization

RSB was generated using a traditional kiln (Jinhefu Ltd, Anshan, China) via slow pyrolysis (400–500 °C) at atmospheric pressure for 24 h; pyrolysis was initiated by burning the feedstock at the bottom of the kiln. The characteristics of RSB are shown in Table 2. Total C, N, S and O were determined by an Elementar Vario Max Analyzer (Vario Micro Cube, Elementar, Germany). Volatile matter was determined according to GB/T 2001-91 (http://www.51zbz.net/biaozhun/61958.html). The surface area was determined by the Brunauer-Emmett-Teller equation (V-Sorb 4800 P, Gold Spectrum Technology, China). The ash content was determined according to GB/T 12496.4-1999 (http://down.foodmate.net/standard/sort/3/5367.html). Micronutrients were analyzed by inductively coupled plasma-atomic emission spectroscopy (AA-7000, Shimazdu, Japan). The pH was determined in a suspension of RSB in water (ratio of 1:10) according to the method of Conyers and Davey (1988)^40^ using a pH instrument (PHS-4CT, Kangyi, China). Water-soluble carbon was determined using a TOC analyzer (3100, Shimazdu, Japan). Water-soluble phenols were determined by using a modified Folin method^41^.

**Table 2:** Physicochemical characteristics and micronutrient concentrations of RSB.

Parameter	Micronutrient concentration (mg/g)		
Physicochemical characteristics			
Total carbon (mg/g)	749.30	Ash	210
Total nitrogen (mg/g)	13.40	Na	2.87
Total sulfur (mg/g)	5.70	Mg	10.36
Total oxygen (mg/g)	112.90	Al	2.31
Extractable potassium (mg/kg)	8.12	Ca	4.98
Water-soluble carbon (mg/g)	2.1	Fe	1.84
Water-soluble phenols (μg/g)	5.9	Pb	0.003
Volatile matter (mg/g)	164.5	Cr	0.11
Surface area (m^2^/g)	8.9	Mn	1.54
pH	7.9	Cu	0.015
color	black	Zn	0.12

### Animal and housing

Specific pathogen-free 5-week-old female Wistar rats weighing 190–210 g (purchased from Lioaning Changsheng Life Sciences Co., Ltd, Benxi, China) were housed in a standard room at a temperature of 22 ± 2 °C and a relative humidity of 53 ± 2% under a 12-h/12-h light-dark cycle. Rats were allowed ad libitum access to chow and water. The Animal Ethics Committee of Shenyang Agricultural University approved this study, and all management and experimental procedures were conducted according to the Guidelines for the Care and Use of Animals of Shenyang Agricultural University.

### Experimental protocols

Following acclimation for 1 week, rats were randomly divided into control and RSB groups of 10 rats per group. RSB was ground, sieved and premixed with ultrapure deionized water and administered to rats by oral gavage at a dose of 1120 mg/kg of body weight (BW) daily for 5 weeks. The control group received an equivalent amount of ultrapure deionized water instead. The gavage volume of 1 mL/100 g of BW was adjusted according to the weight of the rat once per week. The RSB dose was determined based on the optimal dose obtained in piglets in our previous study^14^ using a dose conversion formula for rats according to a pharmacological method^42^. The BW of each rat was assessed once per week throughout the study. At the end of the experiment, all rats were fasted overnight and anesthetized with ether on the 36^th^ d of the experiment. Approximately 1 mL of blood was collected by using the internal canthus vein method and centrifuged (Sartorius-Sigma, Göttingen, Germany) (3000 × g) for 10 min to obtain the serum. Then all the rats were anesthetized with ether and sacrificed by cervical dislocation, and cecal contents of 7 rats (n = 7) were randomly sampled immediately from each group, snap-frozen in liquid nitrogen and stored at −80 °C prior to the analysis of microbial community. Additionally, jejunum and ileum segments were excised to evaluate the histopathology.

### Histopathological evaluation

Two-centimeter segments of the distal jejunum and ileum fixed in 10% formaldehyde solution were dehydrated, paraffin-embedded, sliced and stained with hematoxylin and eosin (H&E) to observe the degree of intestinal villus damage using a biomicroscope (Axio Scope A1; Zeiss, Oberkochen, Germany). Five discontinuous fields were observed for each slice, and 5 villi in each field were measured quantitatively by using ImageJ 1.46r software to analyze villus height (the vertical distance from the intestinal gland to the villus tip), intestinal mucosal thickness (the vertical distance from the villus tip to the bottom), and crypt depth (the vertical distance from the opening of the intestinal gland to the bottom).

### Transmission electron microscopy assay

Distal ileal sections measuring 1 × 1 × 2 mm^3^ were cut and immediately transferred into 2.5% glutaraldehyde and 1% osmium tetroxide for post-fixation. The sections were then embedded in Epon 812 and sliced and double-stained with uranyl acetate and lead citrate to examine ultra-structural changes of the intestinal epithelium with a transmission electron microscope (TEM) (HT-7700; Hitachi, Tokyo, Japan).

### Determination of biochemical parameters

After thawing at less than 4 °C, the serum activity of diamine oxidase (DAO) was analyzed using ELISA test kits (R&D Inc., Los Angeles, CA, USA). All procedures were performed according to the manufacturer’s instructions.

### DNA extraction and PCR amplification for 16S RNA sequencing

DNA was extracted from 0.3 g of cecal content using an E.Z.N.A. Stool DNA kit (D4015, Omega, Inc., USA) in accordance with the manufacturer’s instructions. The V3-V4 region of the prokaryotic small-subunit (16S) rRNA gene was amplified with slightly modified versions of primers 338 F (5′-ACTCCTACGGGAGGCAGCAG-3′) and 806R (5′-GGACTACHVGGGTWTCTAAT-3′)^43^. The 5′ ends of the primers were tagged with specific barcodes for each sample and sequencing universal primers. PCR amplification reactions were performed in a total volume of 25 μL, and the reaction mixtures contained 25 ng of template DNA, 12.5 μL of PCR Premix, 2.5 μL of each primer, and PCR-grade water to adjust the volume. The PCR conditions to amplify the prokaryotic 16 S fragments consisted of an initial denaturation at 98 °C for 30 s, followed by 35 cycles of denaturation at 98 °C for 10 s, primer annealing at 54 °C for 30 s, and extension at 72 °C for 45 s, and then a final extension at 72 °C for 10 min. The PCR products were confirmed by 2% agarose gel electrophoresis. Throughout the DNA extraction process, ultrapure water, rather than a sample solution, was used to exclude the possibility of false-positive PCR results as a negative control. The PCR products were purified by AMPure XP beads (Beckman Coulter Genomics, Danvers, MA, USA) and quantified by Qubit (Invitrogen, Carlsbad, California, USA). The amplicon pools were prepared for sequencing, and the size and quantity of the amplicon library were assessed by an Agilent 2100 Bioanalyzer (Agilent, USA) and with a Library Quantification Kit for Illumina (Kapa Biosciences, Woburn, MA, USA), respectively. A PhiX Control library (V3) (Illumina) was combined with the amplicon library (expected at 30%). The libraries were sequenced with 300PE MiSeq runs, while one library was sequenced using standard Illumina sequencing primers, eliminating the need for a third index read.

### Sequence analysis

Bacterial high-throughput sequencing analysis was performed on an Illumina MiSeq platform according to the manufacturer’s recommendations, provided by LC-Bio. Paired-end reads were assigned to samples based on their unique barcodes and truncated by cutting off the barcode and primer sequences. Paired-end reads were merged using FLASH. Quality filtering of the raw tags was performed under specific filtering conditions to obtain high-quality clean tags according to FastQC (V0.10.1). Chimeric sequences were filtered using Verseach software (V 2.3.4). Sequences with ≥97% similarity were assigned to the same operational taxonomic units (OTUs) by Verseach (V 2.3.4). Representative sequences were chosen for each OTU, and taxonomic data were then assigned to each representative sequence using the RDP (Ribosomal Database Project) classifier. Multiple sequence alignment was performed using PyNAST software to assess differences between the dominant species in different groups and to study the phylogenetic relationships of different OTUs. OTU abundance information was normalized using a standard sequence number corresponding to the sample with the least number of sequences. Alpha diversity was applied to analyze the complexity of species diversity for a sample using 4 indices, Chao1, Shannon, Simpson and OTU number; these indices were calculated with QIIME (Version 1.8.0). Beta diversity analysis was used to evaluate differences between samples in terms of species complexity. Beta diversity was calculated by principal coordinate analysis (PCoA) and cluster analysis using QIIME software (Version 1.8.0).

### Data analyses

Data were statistically analyzed by one-way analysis of variance (ANOVA) using IBM SPSS statistical software, version 22.0, and differences among groups were compared using Duncan’s multiple test. The results were expressed as the mean ± SD, and a 5% level of probability was considered significant for all analyses.

## Electronic supplementary material

Supplementary Information
