# Supplementary material for: Rice straw biochar as a novel niche for improved alterations to the cecal microbial community in rats
Source: Sci Rep. 2018 Nov 6;8:16426. doi: 10.1038/s41598-018-34838-1 (PMC6219602; doi:10.1038/s41598-018-34838-1)
Supplement: Supplementary file 1 — Supplementary Information [file 41598_2018_34838_MOESM1_ESM.docx]

**Supplementary information**

**Rice straw biochar as a novel niche for improved alterations to the cecal microbial community in rats**

Jie Han^1,2^, Jun Meng^2,*^, Shuya Chen^1^, Chuang Li^1^ & Shuo Wang^3^

^1^Key Laboratory of Zoonosis of Liaoning Province, College of Animal Science and Veterinary Medicine, Shenyang Agricultural University, Shenyang 110866, China.

^2^Liaoning Biochar Engineering & Technology Research Center, Shenyang Agricultural University, Shenyang 110866, China.

^3^Testing and Analysis Center, Shenyang Agricultural University, Shenyang 110866, China.

Corresponding author: Jun Meng

E-mail: [mengjun1217@163.com](mailto:mengjun1217@163.com)


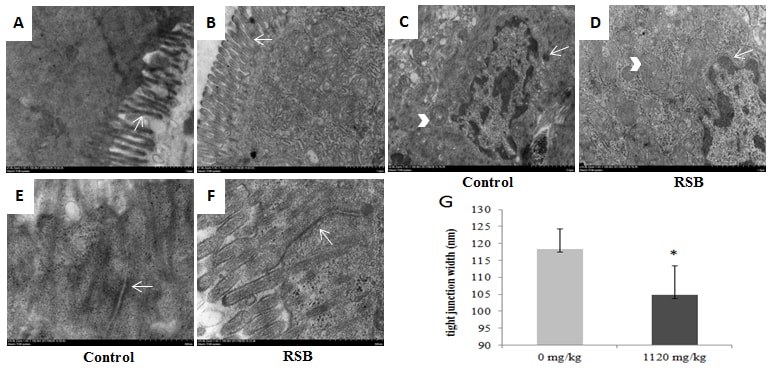


**Figure S1.** Effects of RSB administration on the ultra-structure of the gut epithelium.

(A, B) Representative TEM images of the ultra-structure of the microvilli in the ileal tissue, with arrows indicating the location of the microvilli; (C, D) representative TEM images of the ultra-structure of the nucleus and a mitochondrion in gut epithelial cells in the ileal mucosa, with arrows indicating the location of the nucleus and arrowheads indicating the mitochondrion; (E, F) representative TEM images of the ultra-structure of tight junctions (TJs) in the ileal epithelium, with arrows indicating the location of the TJ; (G) TJ width in the ileal epithelium. Values are the mean±SD, *indicates a significant difference from the control group (n=3).

**Table S1.** Statistical summary of the raw data. C2~C7 indicate rats from control group; R1~ R7 indicate rats from RSB group.

| Sample name | *Raw reads* | Valid reads | Q20% | Q30% | GC% |
| --- | --- | --- | --- | --- | --- |
| C2 | *46498* | 45582 | 96.28 | 87.58 | 53.08 |
| C3 | *53362* | 51486 | 95.80 | 86.06 | 52.62 |
| C4 | *112798* | 109638 | 95.53 | 85.41 | 53.08 |
| C5 | *49154* | 48478 | 89.44 | 72.56 | 53.59 |
| C6 | *45852* | 45422 | 95.34 | 84.96 | 52.62 |
| C7 | *37270* | 36428 | 94.71 | 83.50 | 54.18 |
| R1 | *50930* | 49556 | 96.46 | 88.21 | 53.50 |
| R2 | *126162* | 124402 | 95.36 | 85.14 | 54.11 |
| R3 | *40158* | 39140 | 94.17 | 82.08 | 53.88 |
| R4 | *77110* | 70600 | 96.15 | 87.92 | 53.91 |
| R5 | *121104* | 118542 | 96.20 | 87.83 | 53.87 |
| R6 | *77148* | 74642 | 96.06 | 87.71 | 54.72 |
| R7 | *67498* | 65416 | 96.15 | 87.88 | 54.02 |
| Total | *905044* | 879332 |  |  |  |
| Average | *69618* | 67640 |  |  |  |
